# 

**Published:** 1869-09-15

**Authors:** 


### *CONTENTS.*

COMMUNICATIONS.
- Tic Douloureux—Two Cases ..... 525
- A Uterine Polypus removed with the aid of Ergot ..... 530
- Poisoning by Belladonna ..... 531

FOREIGN ITEMS.
- Investigations into the Intimate Structure of the Pancreas ..... 532
- Bibliographical—The Pathogenesis of Hæmorrhage ..... 533

SOCIETY REPORTS.
- Chicago Medical Society ..... 535

EDITORIAL.
- Vaccination ..... 540

SELECTION.
- What are the Merits of our Chinese Physicians ..... 542

BOOK NOTICES ..... 546

NEWS ITEMS ..... 548

OBITUARY ..... [illegible]

LOOT ..... 551

